# Plant Genetic Archaeology: Whole-Genome Sequencing Reveals the Pedigree of a Classical Trisomic Line

**DOI:** 10.1534/g3.114.015156

**Published:** 2014-12-18

**Authors:** Patrice A. Salomé, Detlef Weigel

**Affiliations:** Department of Molecular Biology, Max Planck Institute for Developmental Biology, D-72076 Tübingen, Germany

**Keywords:** trisomic line, circadian period, flowering time, *GIGANTEA*, deep-sequencing

## Abstract

The circadian oscillator is astonishingly robust to changes in the environment but also to genomic changes that alter the copy number of its components through genome duplication, gene duplication, and homeologous gene loss. While studying the potential effect of aneuploidy on the *Arabidopsis thaliana* circadian clock, we discovered that a line thought to be trisomic for chromosome 3 also bears the *gi-1* mutation, resulting in a short period and late flowering. With the help of whole-genome sequencing, we uncovered the unexpected complexity of this trisomic stock’s history, as its genome shows evidence of past outcrossing with another *A. thaliana* accession. Our study indicates that although historical aneuploidy lines exist and are available, it might be safer to generate new individuals and confirm their genomes and karyotypes by sequencing.

The anticipation of daily transitions like dawn and dusk by a circadian clock provides a fitness advantage in cyanobacteria, plants, fungi, and animals (Dodd *et al.* 2005; Green *et al.* 2002; Xu *et al.* 2011; Yerushalmi *et al.* 2011). Although the circadian clock comprises a highly interconnected network of transcriptional activators and repressors, this adaptation to a rhythmic environment has been maintained while the expansion of gene families and whole-genome duplication or triplication events have altered the balance between different clock components. With the advent of genome-sequencing platforms, we are now in an ideal position to study the evolutionary trajectory of clock gene families, including gene copy number variation, and the birth and death of new copies. Because of its network properties, the circadian clock constitutes a great model system in which to test the gene balance hypothesis. Indeed, Albert Blakeslee observed already in the early 1920s that plants with three copies of a single chromosome are generally less vigorous and fertile than polyploid lines in which all chromosomes have been triplicated, leading to the formulation of genetic (or gene) balance (Blakeslee *et al.* 1920; Blakeslee 1921). In *Arabidopsis thaliana*, some circadian mutants like the *toc1-1* allele were first thought to exhibit dosage sensitivity (Millar *et al.* 1995), but this was later attributed to a dominant-negative mutation in the repressor domain of the protein (Gendron *et al.* 2012). Most *A. thaliana* clock mutations have recessive effects, indicating that a single gene copy is sufficient to maintain period length close to 24 hr, and thus demonstrates some dosage compensation. However, increasing gene copy number for *TOC1* (Mas *et al.* 2003) or Z*TL* (Somers *et al.* 2004) changes period length in a dose-dependent manner, suggesting that network stoichiometry is important. A related phenomenon has been observed in mice, where segmental trisomy 16 shortens the clock period in constant darkness, demonstrating that aneuploidy may lead to alterations in circadian rhythms (Ruby *et al.* 2010). Following whole-genome duplication, clock genes in *Brassica rapa* are more likely to be retained compared with their neighboring genes or random and control genes (Lou *et al.* 2012). Properly functioning circadian clocks confer fitness benefits (Dodd *et al.* 2005; Green *et al.* 2002), putting strong selective pressure on clock genes to be maintained in a stoichiometrically balanced fashion. We wished to determine what circadian phenotypes might be observed in trisomic lines generated by George Redeí and others in the 1960s and available from the *Arabidopsis* stock centers (Redeí 1962; Steinitz-Sears 1963). Although a stock presumed to be trisomic for chromosome 3, CS3227/N3227, had a shorter period, the cause was found to be a 5-bp deletion in the *GIGANTEA* gene found also in one of Redeí’s other stocks, *gi*-1 (Redeí 1962; Park *et al.* 1999). Whole-genome sequencing revealed that the supposedly trisomic stock is an introgression line, with one fifth of its genome coming from an Estland/Estonia (Est)-like accession. An accession from Est was one of ten *Arabidopsis* accessions widely studied by Friedrich Laibach (Laibach 1943) and later George Redeí (Redeí 1962), making it reasonable to deduce that this strain arose through inadvertent outcrossing.

## Materials and Methods

### Plant material and growth conditions

Seed stocks were ordered from stock centers: CS3227 [from Arabidopsis Biological Resource Center (ABRC)] and N3227 [from Nottingham *Arabidopsis* Stock Centre (NASC)], *gi*-1, *gi*-2, *gi*-3 (N3123, N3124, and N51 from NASC). The transfer DNA insertion allele *gi*-201 has been described (Martin-Tryon *et al.* 2007) and is presumed to be a null allele. Mutants *ft*-10 and *fkf*-1 came from laboratory stocks. The *TOC1:LUC* luciferase reporter line in the Columbia-2 (Col-2) background was described previously (Salomé and McClung 2005). All mutants and CS3227 were crossed to this line; mutant seedlings homozygous for the mutation in question (*toc*1-101, *gi*-1, *gi*-2, *gi*-201, *lhy*-20) were selected based on circadian or flowering time phenotypes. We did not observe any natural variation arising from crossing the Col-2 accession (harboring the luciferase (LUC) reporter) to the accessions Col-0 (background of *toc1*-101, *lhy*-20 and *gi*-201) or Col-1 (backgrounds for *gi*-1 and *gi*-2). Flowering time of all genotypes was scored as number of leaves (rosette and cauline) as well as days to flowering, in long days (16-hr light:8-hr dark, LD) at 23°, with a relative humidity of 65%; at least 20 plants were grown for each genotype.

### Cotyledon movement

Seeds were surface-sterilized by the vapor-phase method (Clough and Bent 1998) and plated on Murashige and Skoog (MS) medium supplemented with 2% sucrose and 0.8% agar. After stratification for 3 d at 4° in the dark, plates were released and seedlings allowed to grow for 5 d under a photoperiod of 12-hr light: 12-hr dark, at 23°. On day 6, individual seedlings were transferred to 24-well cloning plates on a cube of growth medium and released into constant light at 23°. Cotyledon position was captured with surveillance cameras over 7 d. Postrun analysis was conducted with the help of the Kujata software package, and circadian parameters estimated by Fast-Fourier transform as previously described (Salomé and McClung 2005).

### Circadian analysis of LUC reporters

Surface-sterilized seeds were plated on MS medium containing 1% sucrose and 0.5% agar and stratified for 2 d at 4° in the dark. After release in 23°, seedlings were entrained by light-dark cycles (16-hr light: 8-hr dark) for 7 d, and were then transferred to 96-well plates. Each well contained 200 µL of MS medium supplemented with 1% sucrose and 30 µL of 2.5 mM luciferin, potassium salt (Biosynth, Postfach, Switzerland). Plates were subjected to another entraining cycle and then moved into constant light and temperature for 5 d for luciferase activity measurements on a Perkin Elmer Topcount microplate luminescence counter. Circadian parameters were extracted from time-course data by Fast-Fourier transform as previously described (Salomé and McClung 2005). All experiments were performed at least three times (n = 12−24).

### Hypocotyl length measurements

Surface-sterilized seeds were plated along the diagonal of Petri dishes containing full-strength MS medium supplemented with 1% sucrose and 0.8% agar. Plates were placed at 4° for 3 d in the dark before being released in short days for 6 d. Images of plates were acquired on a flat-bed scanner in transparent mode alongside a ruler. Hypocotyl length was measured with ImageJ (http://rsbweb.nih.gov/ij/).

### Next-generation sequencing and data analysis

Genomic DNA from *gi*-1, *gi*-2, Est-1 from the Max Planck Institute (MPI; CS22683), Est from the Salk Institute (CS67485), and CS3227 was isolated with the QIAGEN Plant Mini kit. A total of 1 µg of genomic DNA was used as starting material for Illumina’s TruSeq PCR-free library preparation according to manufacturer’s instructions for 350-bp insert size (Illumina, San Diego, CA). Final libraries were quantified by quantitative polymerase chain reaction (qPCR Kapa Biosystems, Boston, MA) and confirmed by Bioanalyzer (Agilent Technologies, Santa Clara, CA) and sequenced on a HiSeq2000 instrument with 2 × 101-bp reads. Short reads were processed with the SHORE pipeline (Schneeberger *et al.* 2009); after filtering of reads that did not map to the Col-0 reference genome, genome-wide coverage was about 20x for *gi*-1, 28x for *gi*-2, and 24x for CS3227, and more than 30x for Est accessions. High-quality single-nucleotide polymorphisms (SNPs) and small deletions between Col-0 and other genotypes were derived from *shore consensus* by the use of a scoring matrix optimized for identifying homozygous positions that differ from the reference genome (scoring_matrix_hom.txt). The resulting SNP lists were compared in R (The R Foundation for Statistical Computing 2013) with the help of the *merge* and *intersect* functions.

## Results

### The genetic stock CS3227 has a short period

**Figure 1:**
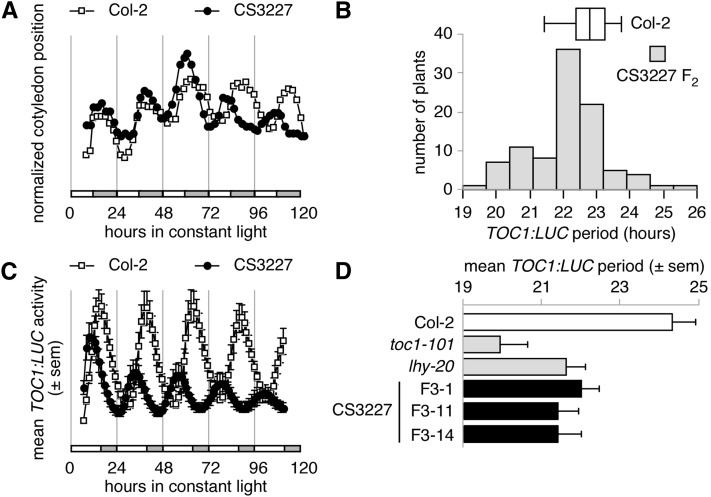
CS3227 has a short period. Seedlings were grown under 12L:12D cycles for 5 (A) or 7 (B−D) d before release into constant light for recording of circadian parameters. (A) Cotyledon movement for Col-2 and CS3227 seedlings. (B) Segregation of the short period phenotype in an F_2_ population derived from a cross between CS3227 and a *TOC1:LUC* reporter in the Col-2 background. (C) Mean *TOC1:LUC* activity for Col-2 and CS3227 seedlings after release into constant light and temperature. (D) Mean *TOC1:LUC* period for Col-2, CS3227 F_3_ lines and the short period mutants *lhy-*20 and *toc1-*101, under the same conditions as (B) and (C). All data shown as means ± SEM (n = 12−24).

Seeds were obtained for a trisomic stock carrying three copies of chromosome 3 from the *Arabidopsis* Biological Resource Center. This stock, referred to herein as CS3227 (N3227 from NASC), displayed a short period when assayed by cotyledon movement (Figure 1A). This phenotype was observed in all seedlings from the original stock received from ABRC, as well as all subsequent generations derived from the original stock. This phenotype did not segregate in any of these populations (representing at least three generations of seed-to seed propagation). Plants trisomic for chromosome 3 are expected to exhibit yellow-green rosette leaves and reduced fertility (www.arabidopsis.org), but we did not select progeny based on these phenotypes at any point, suggesting that the short circadian period was not linked to a compromised dosage of clock genes located on chromosome 3 but rather to an independent genetic defect that was fixed in this stock.

We introduced a *TOC1:LUC* luciferase reporter (in the Col-2 background; Salomé and McClung 2005) into CS3227 by genetic crossing. As shown in Figure 1 B and C, the short period phenotype segregated as a simple, recessive mendelian trait, further confirming that the circadian defect was not caused by aneuploidy. We selected several F_2_ seedlings with a short period and confirmed their phenotype in their progeny (Figure 1D and Table 1). Free-running period in CS3227 was 2 hr shorter than the stated parental line Col-2: 21.9 ± 0.1 hr for F_3_ lines (n = 12) *vs.* 24.2 ± 0.1 hr. CS3227 shortened period as much as *lhy*-20 (21.6 ± 0.1 hr, n = 12), a null allele of the *LATE ELONGATED HYPOCOTYL* gene, but remained longer than a strong allele of the *TIMING of CAB2 1* gene (*toc1*-101: 19.7 ± 0.2 hr, n = 12; Figure 1D and Table 1).

**Table 1:** Circadian parameters of genotypes

Genotype	Period ± SEM	Amplitude ± SEM	RAE*^a^* ± SEM	n
Col-0	24.2 ± 0.1	19,435 ± 1653	0.17 ± 0.01	24
*gi-*1	21.9 ± 0.2	6222 ± 770	0.41 ± 0.03	23
*gi-*2	25.0 ± 0.4	3154 ± 404	0.31 ± 0.03	22
*gi-*201	24.1 ± 0.1	1878 ± 179	0.16 ± 0.02	12
CS3227	21.2 ± 0.2	6111 ± 945	0.27 ± 0.02	12
Col-0	24.3 ± 0.1	15,580 ± 1785	0.14 ± 0.01	23
CS3227	22.0 ± 0.1	4909 ± 1004	0.20 ± 0.02	12
*lhy-*20	21.6 ± 0.1	3628 ± 282	0.12 ± 0.01	12
*toc1-*101	19.9 ± 0.1	980 ± 115	0.20 ± 0.03	23

a RAE refers to relative amplitude error, with an RAE of zero corresponding to a perfect sine wave, while RAEs closer to 1 denote weaker rhythms.

### The genetic stock CS3227 is late flowering

**Figure 2:**
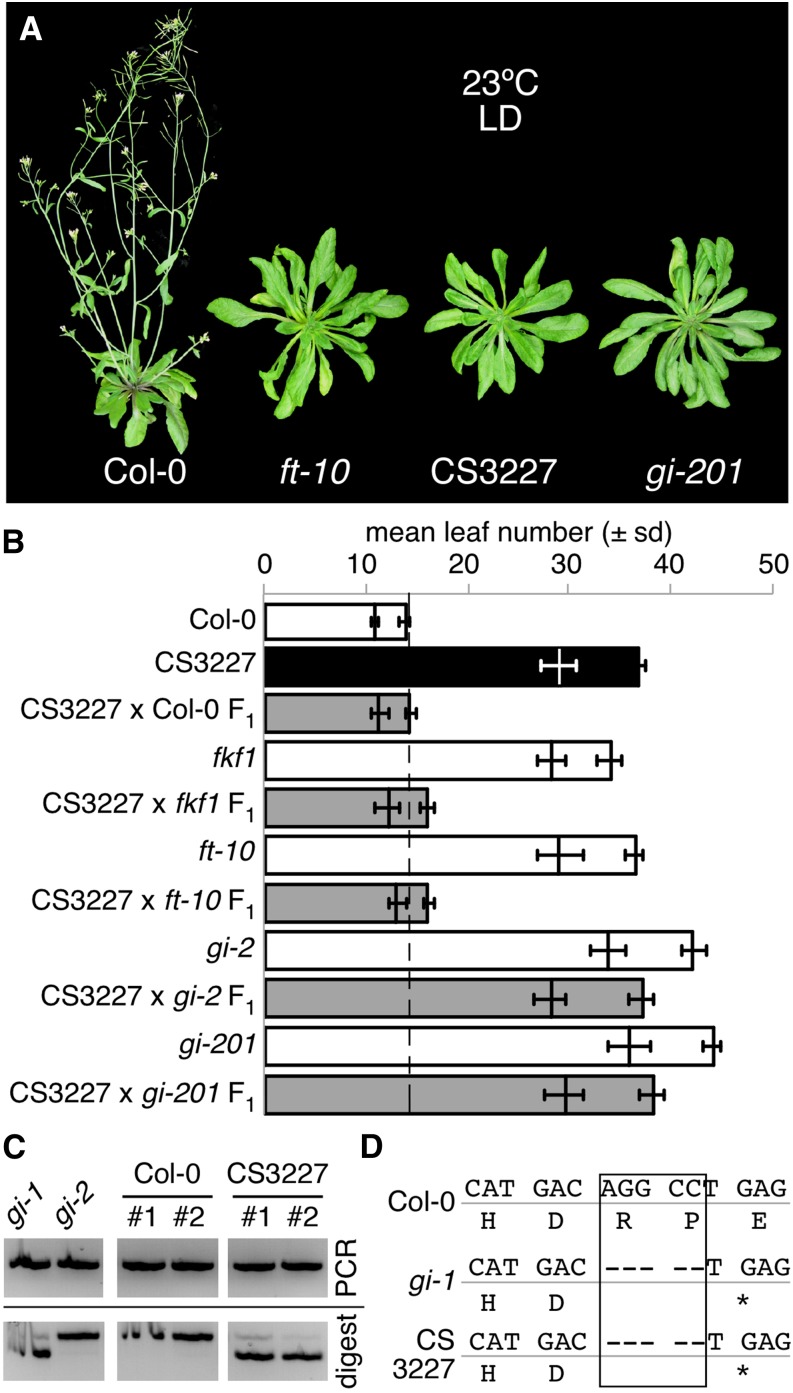
CS3227 is late flowering and carries the same deletion as *gi*-1. (A) Representative adult plants for Col-0, *ft*-10, CS3227, and *gi*-201 strains grown for 4 wk in long days (LD) at 23°. (B) Mean rosette and cauline leaf number in F_1_ plants from crosses between CS3227 and various late flowering mutants along with parental controls. (C) CAPS analysis of the genetic lesion found in the *GIGANTEA* gene in CS3227. Two independent DNA preparations were tested for Col-0 and CS3227 and are referred to as #1 and #2. PCR products were first precipitated to remove excess salts, as *Psy*I digest performed poorly when directly added to the PCR. Only the larger digest product is shown for *gi-1* and CS3227 samples. PCR, polymerase chain reaction. (D) Alignment of genomic sequences for the *GI* locus in Col-0, *gi*-1, and CS3227, around the 5-bp deletion detected in CS327. The asterisk denotes the position of the premature stop codon introduced by the deletion.

An additional phenotype, delayed flowering, was observed in CS3227 plants when grown in long days (16-hr light) at 23°. The delay in initiation of the reproductive phase was similar to the other late flowering mutants *fkf1*, *ft*, and *gi* (Figure 2 A and B). Late flowering and short period phenotypes of CS3227 always cosegregrated (Figure 2B), indicating that they were likely caused by the same genetic lesion. We tested whether CS3227 was allelic to known late flowering mutants by crossing CS3227 to *fkf1*, *ft*-10, *gi*-2, and *gi*-201. Only F_1_ plants from crosses between CS3227 and both *gi* alleles flowered late, suggesting that *GIGANTEA* might carry a lesion in the CS3227 stock.

Mapping of the short period phenotype in an F_2_ population derived from a cross between CS3227 *TOC1:LUC* and the L*er* accession independently confirmed strong linkage to the top arm of chromosome 1, where *GI* is located. Targeted sequencing of the *GI* locus in CS3227 revealed a 5-bp deletion near the 3′ end of the gene, resulting in the introduction of a premature stop after amino acid 1002. The deletion introduced a *Psy*I restriction site in *GI^CS3227^*, and allowed an independent confirmation with a CAPS marker (Glazebrook *et al.* 1998). PCR amplification followed by *Psy*I digestion yielded two bands of the expected size for CS3227 (Figure 2C). Col-0 and *gi*-2 products did not carry the restriction site and were thus not cleaved by the enzyme. However, *gi*-1, which has exactly the same mutation (Park *et al.* 1999) as we found in CS3227, showed the same restriction pattern as CS3227 samples (Figure 2 C and D). We reordered the seed stock for CS3227 from NASC and found that all plants were homozygous for the *gi-*1 deletion (Table 2). We therefore concluded that the late flowering and short period phenotypes of CS3227 were caused by a small deletion in *GI*, which is identical to the *gi*-1 lesion.

**Table 2:** Frequencies of phenotypes and genotypes observed in a N3227 stock, obtained from NASC

	Observed	Expected*^a^*	% Observed	*P* Value
Late flowering	106	29−30	100	0
*gi-1* / *gi-1* genotype	106	29−30	100	0

NASC, Nottingham *Arabidopsis* Stock Centre.

a Transmission of the trisomic copy of chromosome 3 is based on Steinitz-Sears, 1963 (5 trisomics from 18 offspring, frequency = 27.8%, Steinitz-Sears 1963).

### CS3227 behaves like *gi-*1 for circadian phenotypes

**Figure 3:**
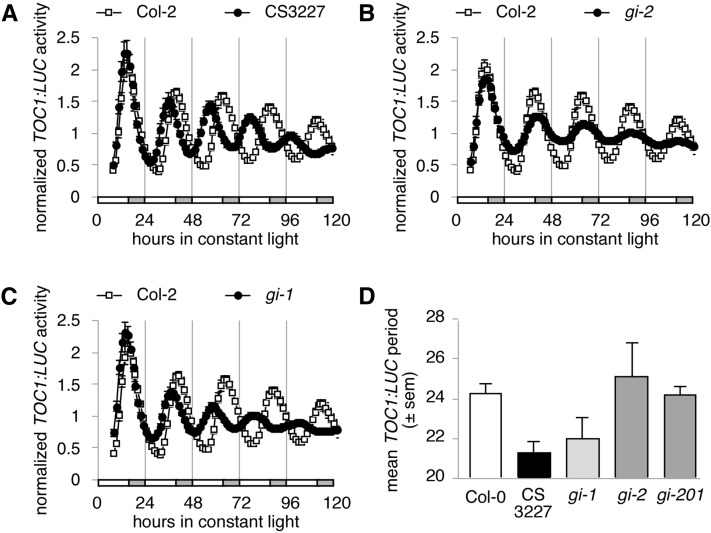
Comparison of CS3227 with other *gi* alleles for circadian period. All seedlings were grown under 12L:12D cycles for 7 d before being released into constant light to record circadian parameters. Mean normalized *TOC1:LUC* activity for Col-2 and CS3227 (A), *gi*-2 (B), and *gi*-1 (C) after release into constant conditions. (D) Mean *TOC1:LUC* period for seedlings shown in (A−C). All data shown as means ± SEM (n = 12−24).

**Figure 4:**
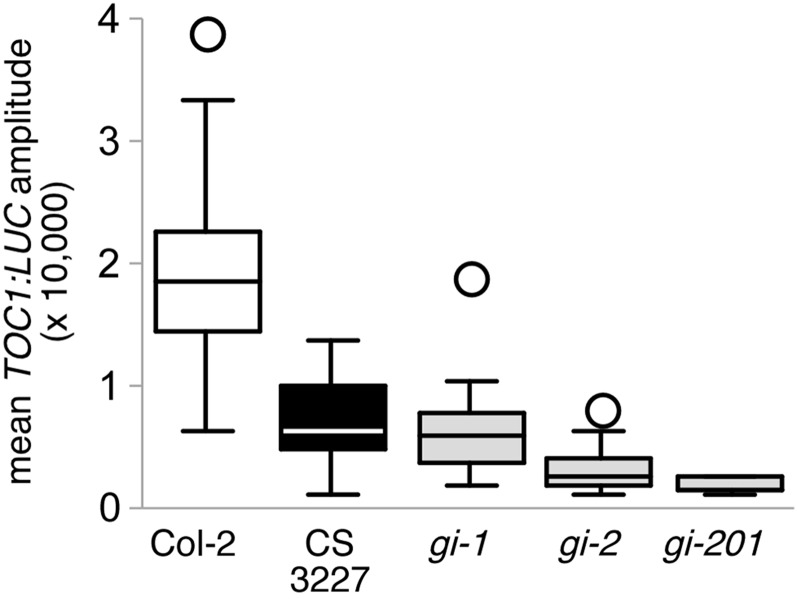
Comparison of CS3227 with other *gi* alleles for circadian amplitude. Mean *TOC1:LUC* amplitude for Col-2 and several *gi* alleles. Data are shown as box and whiskers plot from at least 24 seedlings.

In light of the molecular lesion found in CS3227, we re-examined the circadian defects present in CS3227 alongside the weak *gi* allele *gi*-1 and the strong alleles *gi*-2 and *gi*-201. Both CS3227 and *gi*-1 displayed a similar short period, while the strong *gi* alleles did not affect period length significantly (Figure 3). Circadian amplitude of the reporter was markedly decreased in all *gi* mutants and in CS3227, with strong *gi* alleles having a stronger effect (with values about 10–14% of wild type, Figure 4. The same reporter was introgressed from Col-2 into all mutant backgrounds, allowing direct comparison of amplitudes). Again, *gi*-1 and CS3227 behaved in a similar fashion regarding circadian amplitude, which reached about 40% of wild-type values. The CS3227 genetic stock therefore exhibited all circadian defects known to occur in the *gi*-1 allele.

### CS3227 differs from *gi-1* for petiole and hypocotyl elongation

**Figure 5:**
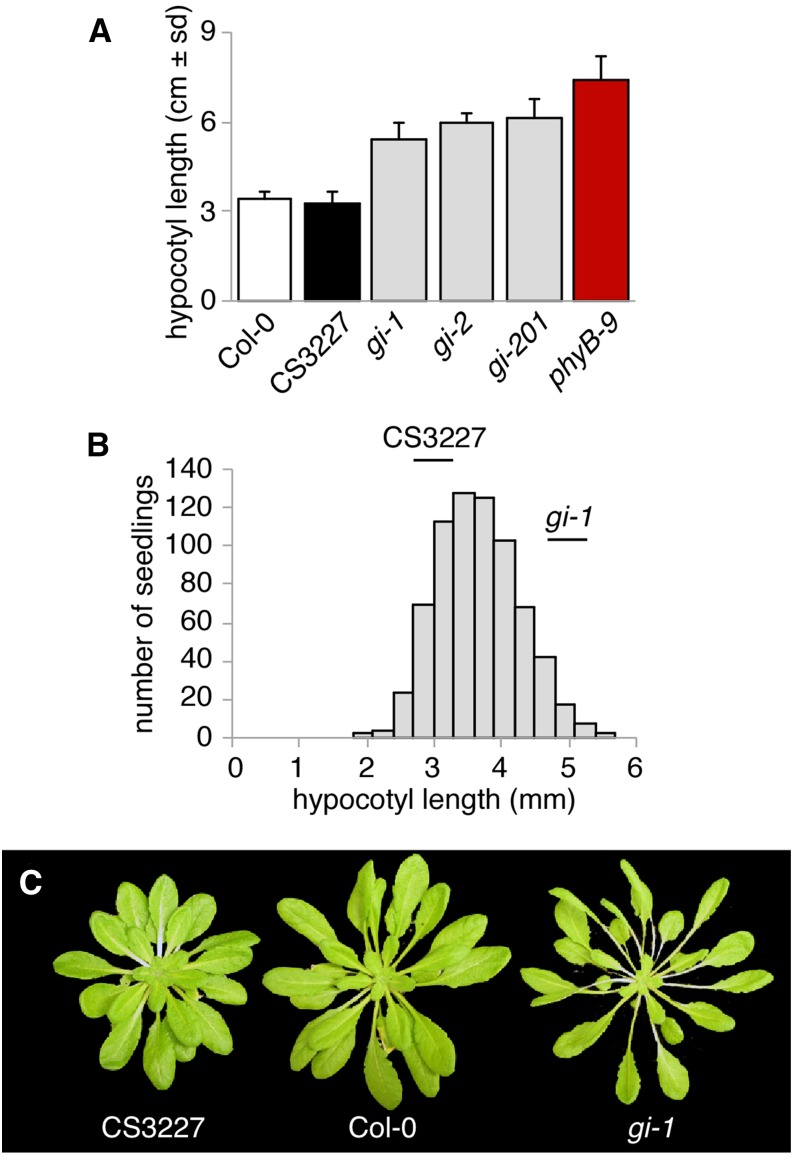
CS3227 carries modifiers that suppress *gi*-induced petiole and hypocotyl elongation. (A) Mean hypocotyl length for Col-0, CS3227, and several *gi* alleles grown for 6 d in short days at 23°. (B) Distribution of hypocotyl length in an F_2_ population derived from a cross between CS3227 and Col-0. (C) Representative adult Col-0, *gi*-1, and CS3227 plants grown for 8 wk in short days at 23°.

Not all aspects of CS3227 aligned with the phenotypes described for *gi*-1. *GI* loss of function alleles are characterized by elongated hypocotyls in red light and white light. We therefore measured hypocotyl length in Col-0, CS3227, and several *gi* alleles when grown in white light and short days. As expected, all *gi* alleles (*gi*-1, *gi*-2, and *gi*-201) had long hypocotyls under these conditions (mean length ~6 mm; with the Col-0 wild-type strain being about 3 mm in length) but remained slightly shorter than the red light photoreceptor mutant *phyB-9* (Figure 5A). In contrast, CS3227 displayed a shortened hypocotyl that was comparable to Col-0. The long hypocotyl characteristic of a *gi*-1 mutant was recovered in an F_2_ population derived from a cross between CS3227 and Col-0, indicating that CS3227 also carried a modifier locus (or loci) that suppressed the long hypocotyl resulting from loss of *GI* function (Figure 5B).

Petiole elongation is strongly promoted in *gi* mutants when grown in short days, as seen for *gi*-1 adult plants (Figure 5C). Petioles in CS3227 were much shorter, and were comparable to those seen in Col-0 (Figure 5C). We concluded that the CS3227 genetic stock carried both the *gi*-1 mutation (a 5-bp deletion) as well as a modifier locus (or loci) acting specifically in GI-dependent control of cell elongation. These results also alleviate potential concerns about seed contamination or simple mislabeling of CS3227, as it clearly displayed a number of phenotypes not seen in *gi*-1.

### Deep-sequencing of CS3227 reveals an unexpected pedigree

CS3227, *gi*-1, and *gi*-2 were isolated by George Redei in the early 1960s. That both CS3227 and *gi*-1 carry an identical 5-bp deletion raised the possibility of a shared genetic history of the two strains. We therefore sequenced the genomes of *gi*-1, *gi*-2, and CS3227 with the Illumina PCR-free DNA TruSeq protocol to identify polymorphisms relative to Col-0 in these genetic stocks and test for relatedness. SNP numbers relative to the Col-0 reference genome sequence are summarized in Table 3. As expected, *gi*-1 and gi-2 shared most of their SNPs, distributed along all 5 chromosomes. SNP numbers were low, consistent with the genetic background in which these two mutants were originally isolated (Col-1, which later gave rise to the commonly used Col-0 stock). A comparison with SNPs identified in CS3227 revealed a more complex picture. For much of the genome, CS3227 had 10−200 times more SNPs per chromosome than *gi*-1 or *gi*-2, but SNP density was distributed unevenly, with large-scale blocks of dense and sparse SNPs (Supporting Information, Figure S1, Figure S2, and Table 3). Chromosome 5 was an exception, with very few SNPs, many of which were shared with *gi*-1 and *gi*-2, presumably due to their common original background (Figure S1).

**Table 3:** Summary of SNP numbers identified in *gi*-1, *gi*-2, and CS3227 relative to the reference Col-0

Genotype	chr 1	chr 2	chr 3	chr 4	chr 5
*gi*-1	391	210	251	775	335
*gi*-2	366	206	248	793	346
CS3227	78,415	2506	8857	55,318	488

SNP, single-nucleotide polymorphism.

**Figure 6:**
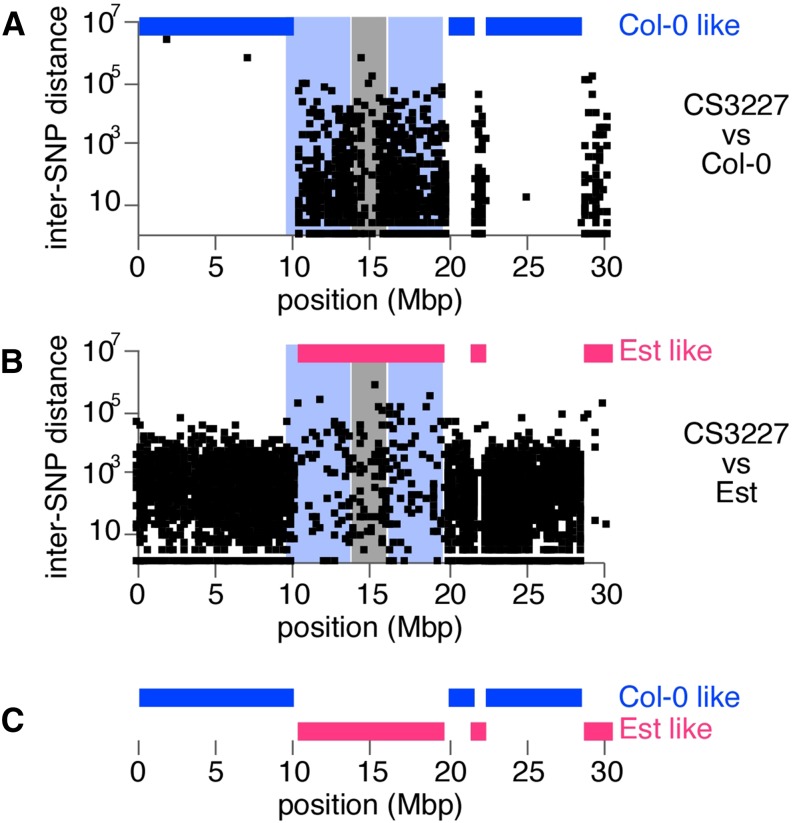
Estland/Estonia (Est) is a likely candidate progenitor for the non−Col-0 genomic regions found in CS3227. The Est accession shared most of its SNPs with the non−Col-0 regions of CS3227, here shown for chromosome 1 but also for chromosomes 2−4. (A) Inter-SNP distance for all SNPs found along chromosome 1 in CS3227 when Col-0 is used as reference. (B) Inter-SNP distance for chromosome 1 of CS3227 with Est as reference genome. (C) Complementary pattern of Col-0−like and Est-like genomic regions along chromosome 1 of CS3227. SNP, single-nucleotide polymorphism.

Although most *Arabidopsis* laboratories currently focus on one of the Col accessions or L*er*, initial *Arabidopsis* research included 10 distinct accessions, all collected and distributed to the community by Friedrich Laibach (Laibach 1943). We therefore compared SNPs of a non−Col-0 portion of chromosome 1 from CS3227 with SNP datasets from seven of the nine non-Col accessions used by George Redeí that have been re-sequenced as part of the 1001 Genomes project (http://1001genomes.org) (Weigel and Mott 2009) and found a match with Est (from Estland/Estonia; Figure S3). Blocks of high similarity between Est and CS3227 coincided with regions with high SNP density relative to Col-0, and were found on all chromosomes except chromosome 5 (Figure 6 and Figure S2).

Samples of the Est accessions have been sequenced at the MPI in Tübingen and at the Salk Institute, but only Est sequenced at the Salk Institute was a very good match to CS3227. We resequenced the Est accessions from Salk (CS67485) and from MPI (CS22683) with the PCR-free DNA TruSeq method used for our initial sequencing. Again, CS3227 was clearly related to Est from Salk, but not to Est-1 from MPI (Figure S3B). Both Est accessions displayed the lesioning/early senescence phenotype characteristic of accessions carrying a hyperactive allele of the *ACCELERATED CELL DEATH 6* (*ACD6*) gene (Todesco *et al.* 2010). Of the two presumptive causal SNPs identified in the Est-1 allele of *ACD6*, only the polymorphism A566N was shared with the Est stock sequenced at the Salk Institute, suggesting that this SNP alone is sufficient to result in early onset senescence. CS3227 plants did not share this phenotype (Figure 2A); in agreement, the genomic location of *ACD6* (about 8.3 Mb on chromosome 4) was of Col origin, although flanked by two Est regions (Figure S2).

Based on haplotype blocks, we identified clear breakpoints for all Est-like regions (Table 4). We counted several Est-like regions on chromosome 1 alone, indicative of at least eight independent crossover events resulting in the observed alternating pattern of Col-0-like and Est-like genomes. Because the average crossover rate for chromosome 1 is about 1.7 after a single meiosis (Salomé *et al.* 2012), this observation is consistent with an outcrossing event followed by many generations of selfing, leading to a pattern typical for recombinant inbred lines.

**Table 4:** Predicted Est-like intervals in CS3227

Chromosome	Left Border, bp	Right Border, bp
1	10,180,611	19,875,136
	21,543,989	22,196,062
	28,423,595	30,427,017
2	0	302,692
3	1,732,545	4,274,768
	4,289,162	4,622,728
4	0	6,215,838

Est, Estland/Estonia.

**Figure 7:**
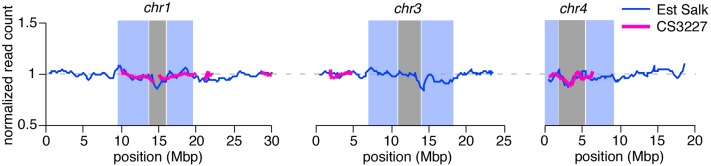
The sequenced CS3227 stock is not trisomic. Normalized read count (over all chromosomes) for Estland/Estonia (Est) Salk (blue line) and the Est-like genomic regions for CS3227 (magenta line).

Finally, we wished to confirm that our CS3227 stock was no longer carrying a third copy of chromosome 3. We therefore plotted normalized read support for all identified Est-like SNPs for CS3227. As shown in Figure 7, read counts for CS3227 were even and showed a pattern very close to that seen in Est from Salk. Importantly, normalized read count for all chromosomes was close to 1, including chromosome 3, indicating that any trisomic history in our stock had been lost prior to the generation we sequenced.

## Discussion

We initially set out to characterize what effect additional chromosomes might have on the *A. thaliana* circadian clock. When it became clear that we did not have trisomic individuals in our test population, we shifted our attention to line CS3227, initially described as being trisomic for chromosome 3, which exhibited a number of interesting phenotypes rarely seen in combinations in other *A. thaliana* mutants: a short circadian period (Figure 1 and Figure 3) and late flowering in long days (Figure 2A). We mapped the causal locus for these phenotypes and found that CS3227 harbored the *gi*-1 mutation, a 5-bp deletion in the *GIGANTEA* gene (Figure 2, B and C). Both *gi*-1 and CS3227 were isolated in the 1960s by George Redeí, anchoring the two stocks to a common origin.

Whole-genome sequencing of the CS3227 genome revealed a mosaic of Col-like and Est-like regions for all but chromosome 5, arguing against a simple seed contamination (Figure 6, Figure S2, and Figure S3). We did not observe greater read coverage for chromosome 3 (Figure 7), consistent with a lack of trisomy in our population. A delay in flowering time was not reported for newly synthesized chromosome 3 trisomics (Henry *et al.* 2010), leading us to conclude (i) that this phenotype is specific for CS3227 and the *gi-*1 deletion, and (ii) that chromosome 3 does not contain genes with major roles in the switch to flowering. Indeed, very few genes with major effects on flowering time have been mapped to chromosome 3.

**Figure 8:**
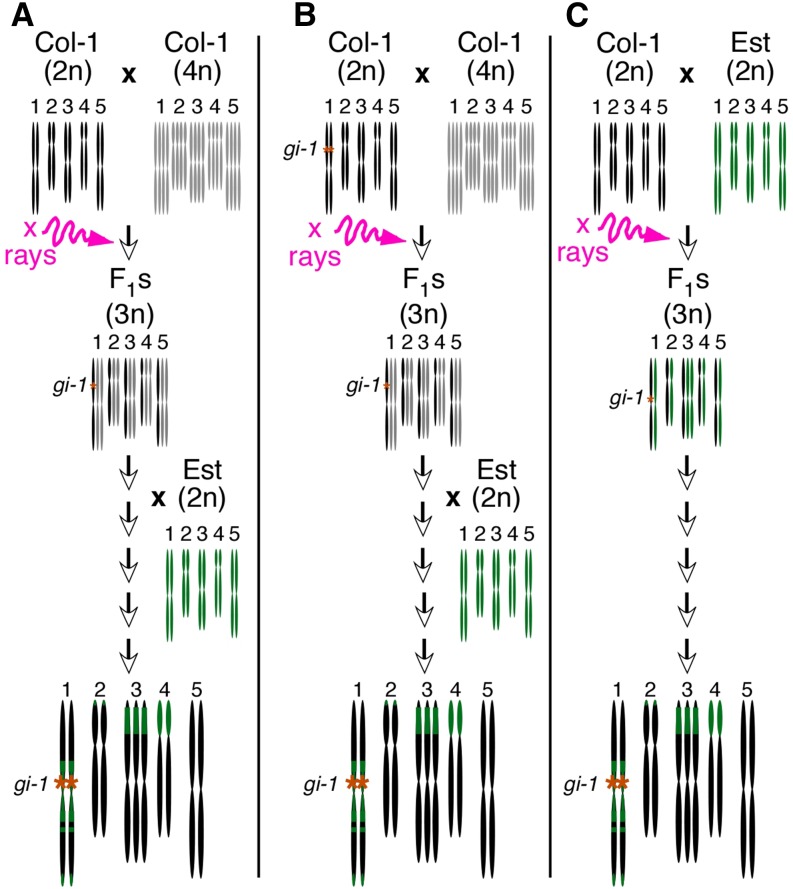
Possible models to explain the genesis of CS3227. In models (A) and (B), the complement of primary trisomics was generated from a cross between a diploid and a tetraploid Col-1 parent. The nonequal segregation of chromosomes in the F_1_s was induced by X-ray irradiation, which may have caused the *gi*-1 deletion (A). Alternatively, *gi-*1 may have been the diploid parent (B). The introduction of the Est-like genome occurred after the isolation of trisomic individuals, either by targeted crossing or by outcrossing to a neighboring Est-like plant. In model (C), two diploid parents, Col-1 and Est-like, were crossed and the F_1_ plants X-ray irradiated. The extent of Est-like genome was subsequently largely lost following backcrosses to Col-1 or by single-seed decent. As in (A), model (C) posits the genesis of *gi-*1 as a consequence of X-ray irradiation of the F_1_ generation.

Even with full genome sequence from our CS3227 stock, it is difficult to reconstruct the exact chain of events that led to its genesis. Three major events must have taken place, the order of which is not clear: the isolation of a trisomic stock for chromosome 3; the introduction of the *gi-*1 deletion; and the introgression of an Est-like genome. Trisomic lines for other chromosomes do not share the *gi*-1 deletion (not shown), arguing against the model depicted in Figure 8B, in which the *gi*-1 mutant was used as diploid parent in the original diploid x tetraploid cross. *gi*-1 and the trisomic lines were both generated by irradiating *A. thaliana* with X rays (Redeí 1962; Steinitz-Sears 1963); it is therefore conceivable that *gi*-1 was isolated in the progeny of the diploid x tetraploid cross (Figure 8, A and C). Although we favor this scenario, we notice that *gi*-1 and CS3227 do not share more SNPs with each other than with *gi*-2. However, ionizing agents do not induce as many point mutations as EMS (Belfield *et al.* 2012) and may therefore not live an easily trackable footprint.

The introduction of the Est-like genome in the ancestral CS3227 stock is more difficult to date. Because of the number of apparent crossovers, most notably on chromosome 1, many generations must separate the initial cross and the current generation we characterized and sequenced. Other trisomic lines were negative for an Est-like genotype at a small number of tested markers, suggesting that the induction of trisomy may have predated the introduction of the Est-like genome. The model depicted in Figure 8A is therefore the most likely scenario to explain how CS3227 came about.

Although the study of changes in circadian parameters caused by aneuploidy merits further attention, it is clear that future efforts should begin with the generation, karyotyping, and phenotyping of aneuploid individuals from genetically defined parental lines. Chromosomal variants can now be easily identified by whole-genome sequencing (Henry *et al.* 2010), thus facilitating the precise identification of aneuploidy lines and the exact chromosome complement of their genomic make-up. It may also be informative to decrease clock gene copy number in a tetraploid background, to observe what possible circadian defects might arise from the loss of a clock gene following whole-genome duplication. Our study also highlights the power of whole genome sequencing to resolve issues arising from mixed genetic stocks, a non-negligible problem in *Arabidopsis* (Anastasio *et al.* 2011).

## Supplementary Material

Supporting Information
