# Supplementary material for: Plant Genetic Archaeology: Whole-Genome Sequencing Reveals the Pedigree of a Classical Trisomic Line
Source: G3 (Bethesda). 2014 Dec 18;5(2):253–9. doi: 10.1534/g3.114.015156 (PMC4321033; doi:10.1534/g3.114.015156)
Supplement: Supporting Information [file supp_5_2_253__index.html]

Plant Genetic Archaeology: Whole-Genome Sequencing Reveals the Pedigree of a Classical Trisomic Line — Supporting Information 

# Plant Genetic Archaeology: Whole-Genome Sequencing Reveals the Pedigree of a Classical Trisomic Line

## Supporting Information for Salomé and Weigel, 2015

**Files in this Data Supplement:**

- Supporting Information - Figures S1-S3 and Tables S1-S5 (PDF, 402 KB)
- Figure S1 - SNP frequency in *gi-1, gi-2* and CS3227. (PDF, 166 KB)
- Figure S2 - SNP distribution in CS3227 is not random. (PDF, 288 KB)
- Figure S3 - SNP sharing between chosen accessions and CS3227. (PDF, 99 KB)
- Table S1 - SNP data for CS3227. (.zip, 867 KB)
- Table S2 - SNP data for EstMPI. (.zip, 4 MB)
- Table S3 - SNP data for EstSalk. (.zip, 4 MB)
- Table S4 - SNP data for *gi*-1. (.txt, 61 KB)
- Table S5 - SNP data for *gi*-2. (.txt, 61 KB)
