# Supplementary material for: Plant Genetic Archaeology: Whole-Genome Sequencing Reveals the Pedigree of a Classical Trisomic Line
Source: G3 (Bethesda). 2014 Dec 18;5(2):253–9. doi: 10.1534/g3.114.015156 (PMC4321033; doi:10.1534/g3.114.015156)
Supplement: Supporting Information [file supp_g3.114.015156_015156SI.pdf]

G3·Genes | Genomes | Genetics

Plant genomic archaeology: whole-genome sequencing reveals the pedigree of a classical trisomic line

Patrice A. Salomé [1,2] and Detlef Weigel

Department of Molecular Biology, Max Planck Institute for Developmental Biology, Spemannstraße 37-39, D-72076 Tübingen, D-72076 Tübingen, Germany

[1] Corresponding author. Email: salome@chem.ucla.edu

[2] Current address: UCLA, Department of Chemistry and Biochemistry, 607 Charles E. Young Drive East, Los Angeles, CA 90095, USA

DOI: 10.1534/g3.114.015156

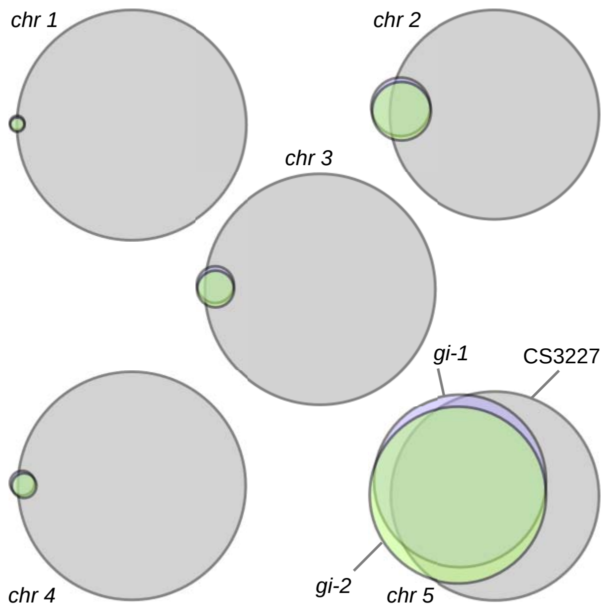

Figure S1 SNP frequency in *gi-1*, *gi-2* and CS3227.
Scaled Venn diagrams for SNP number in the genotypes *gi-1*, *gi-2* and CS3227 for each of the five *Arabidopsis* chromosomes. Note that diagram areas between chromosomes should not be compared, as scaling was not applied between chromosomes. Number of SNPs for each genotype is given in Table 3.

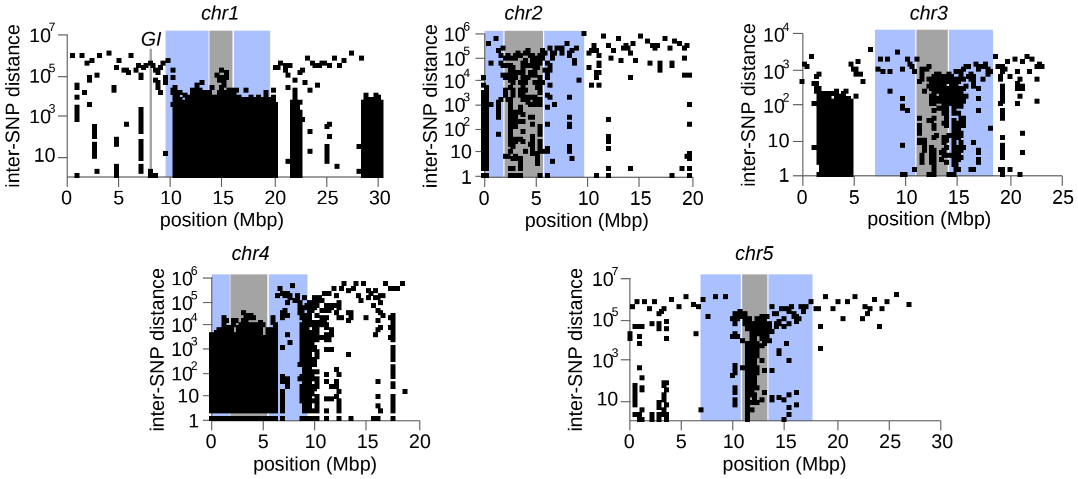

Figure S2 SNP distribution in CS3227 is not random.
Inter-SNP distance between consecutives SNPs is plotted as a function of the mid-position between consecutive SNPs. Clear haplotype blocks appear along all CS3227 chromosomes, with the exception of chromosome 5, which also exhibits the lowest SNP count for this genotype.

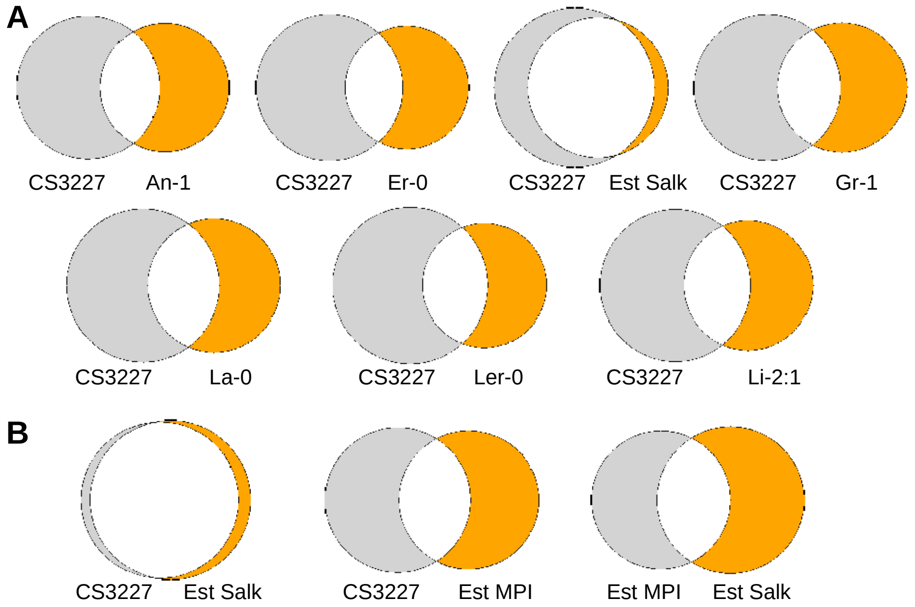

Figure S3 SNP sharing between chosen accessions and CS3227. Note SNP numbers are not corrected for the un-sequenced portion of the compared genomes. All SNP data downloaded from 1001genomes.org on August 2013.

A. SNP sharing between CS3227 and accessions An-1, Er-0, Est, Gr-1, La-0, Ler-0 and Li-2:1 used by George Redeí, for the interval 10-20 Mbp of chromosome 1.

B. SNP sharing between CS3227 and Est accessions (from Salk, CS 67485, or MPI, CS22683), for, after re-sequencing as PCR-free DNA Truseq libraries. Note that SNP numbers are not corrected for the un-mapped portion of the compared genomes.

Tables S1-S5
Available for download at http://www.g3journal.org/lookup/suppl/doi:10.1534/g3.114.015156/-/DC1

Table S1. SNP data for CS3227.
Table S2. SNP data for EstMPI.
Table S3.SNP data for EstSalk.
Table S4. SNP data for *gi*-1.
Table S5. SNP data for *gi*-2.

Tables S1-S5 are provided in Shore output format:
Genotype
Chromosome: chromosome identifier
Position: position of a base in the reference sequence
Reference: nucleotide found in Col-0
Non-reference: nucleotide (or deletion) in the sequenced genome
Quality: Highest base quality supporting the base change, between 0-40. Cut-off was chosen >25.
Support: Number of reads supporting the base change
Concordance: concordance of all short reads overlapping the position
Copy: copy number of the target sequence
