# Supplementary material for: Plant Genetic Archaeology: Whole-Genome Sequencing Reveals the Pedigree of a Classical Trisomic Line
Source: G3 (Bethesda). 2014 Dec 18;5(2):253–9. doi: 10.1534/g3.114.015156 (PMC4321033; doi:10.1534/g3.114.015156)
Supplement: Supporting Information [file supp_g3.114.015156_FigureS1.pdf]

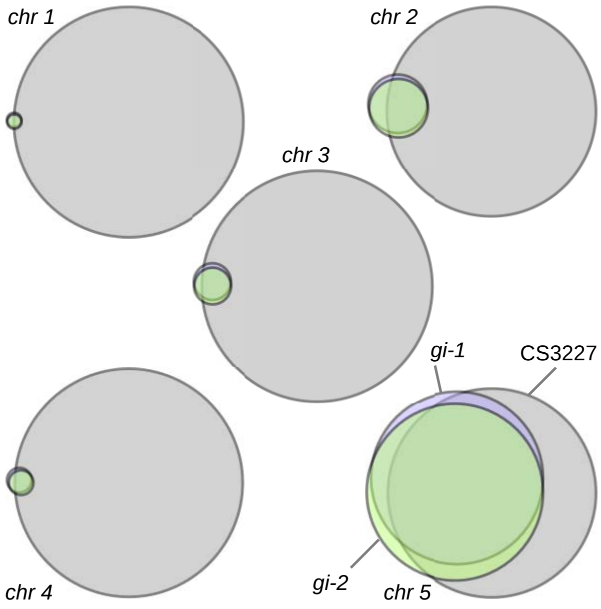

Figure S1 SNP frequency in *gi-1*, *gi-2* and CS3227.
Scaled Venn diagrams for SNP number in the genotypes *gi-1*, *gi-2* and CS3227 for each of the five *Arabidopsis* chromosomes. Note that diagram areas between chromosomes should not be compared, as scaling was not applied between chromosomes. Number of SNPs for each genotype is given in Table 3.
