# Supplementary material for: Plant Genetic Archaeology: Whole-Genome Sequencing Reveals the Pedigree of a Classical Trisomic Line
Source: G3 (Bethesda). 2014 Dec 18;5(2):253–9. doi: 10.1534/g3.114.015156 (PMC4321033; doi:10.1534/g3.114.015156)
Supplement: Supporting Information [file supp_g3.114.015156_FigureS2.pdf]

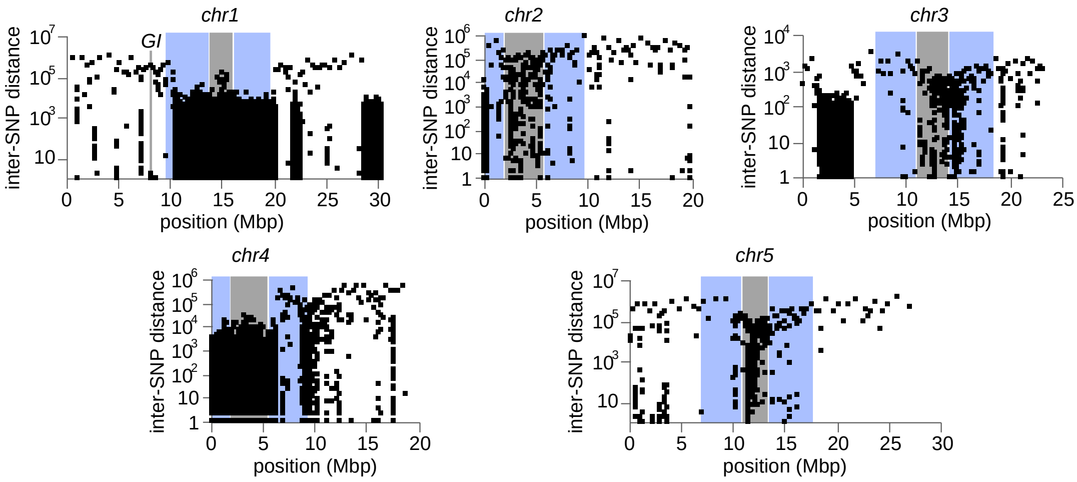

Figure S2 SNP distribution in CS3227 is not random.
Inter-SNP distance between consecutives SNPs is plotted as a function of the mid-position between consecutive SNPs. Clear haplotype blocks appear along all CS3227 chromosomes, with the exception of chromosome 5, which also exhibits the lowest SNP count for this genotype.
