# Supplementary material for: Plant Genetic Archaeology: Whole-Genome Sequencing Reveals the Pedigree of a Classical Trisomic Line
Source: G3 (Bethesda). 2014 Dec 18;5(2):253–9. doi: 10.1534/g3.114.015156 (PMC4321033; doi:10.1534/g3.114.015156)
Supplement: Supporting Information [file supp_g3.114.015156_FigureS3.pdf]

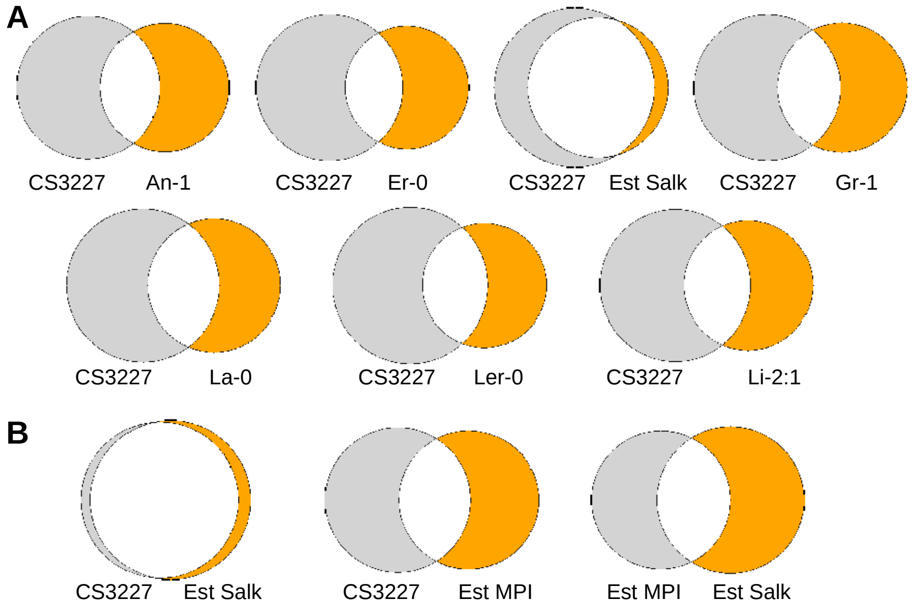

Figure S3 SNP sharing between chosen accessions and CS3227. Note SNP numbers are not corrected for the un-sequenced portion of the compared genomes. All SNP data downloaded from 1001genomes.org on August 2013.

- **A.** SNP sharing between CS3227 and accessions An-1, Er-0, Est, Gr-1, La-0, Ler-0 and Li-2:1 used by George Redeí, for the interval 10-20 Mbp of chromosome 1.
- **B.** SNP sharing between CS3227 and Est accessions (from Salk, CS 67485, or MPI, CS22683), for, after re-sequencing as PCR-free DNA Truseq libraries. Note that SNP numbers are not corrected for the un-mapped portion of the compared genomes.
